# Decentralisation of the Compliance of Anti-tobacco Law in India: The Case of Higher Educational Institutions in New Delhi, India

**DOI:** 10.7759/cureus.41247

**Published:** 2023-07-01

**Authors:** Raja Singh

**Affiliations:** 1 Architecture, School of Planning and Architecture, New Delhi, IND; 2 Centre for Built Environment Policy, Information Sharing and Analysis Center, New Delhi, IND; 3 Built Environment and Public Health, Tathatara Foundation, Bobbili, IND

**Keywords:** public health, team work and public health, proximity of tobacco vendors, public policy, no smoking signboards, smoking control, tobacco cessation, cotpa act 2003

## Abstract

Introduction

This paper studies the decentralised compliance responsibility of India’s tobacco control legislation, called the Cigarettes and Other Tobacco Products (Prohibition of Advertisement and Regulation of Trade and Commerce, Production, Supply and Distribution) Act of 2003, with its rules, which the government has outsourced to higher educational institutions, studied through an example of New Delhi. The study looks into the three most important parameters of decentralised compliance. Two of these require the installation of signboards by higher educational institutions, and the third involves imposing and collecting fines against persons found smoking within the educational institutions. Regarding the boards, the first board is about the warning prohibiting the sale within 100 yards of educational institutions, and the second one prohibits smoking in educational institutions. The study also checks with the educational institutions whether there is a presence of cigarette and tobacco product vendors within 100 yards of the institution, where the sale of such products has been banned by law. The study also found educational activities to create awareness in the institutions for tobacco control and cessation.

Methods

Thirty-six higher educational institutions, including universities, were randomly selected and studied through a unique methodology using India’s transparency law, i.e., the Right to Information Act 2005. The portions of the law, which was direct compliance, or related compliance by the higher educational institution was included in the study. This justified the decentralised responsibility of these higher educational institutions. Applications for information under the transparency law were requested and supplied. Out of the 47 institutions, in which information requests were filed, 36 provided the information under the law. Credible information was provided by the higher educational institutions and this information was collated and interpreted to provide insights into the compliance by the higher educational institutions.

Results

Only 44.4% of the institutions had a board prohibiting the sale of cigarettes and other tobacco products. This was a non-universal compliance by the higher educational institutions. 88.9 per cent had boards prohibiting smoking in the higher educational institutions. Only one out of the 36 had an instance of smoking recorded and collection of fines. While 47.2% reported to not have any instance of smoking and fine collection. Fifty per cent of the institutions had no record of fine collection amounting to defiance of the law. 75 per cent of institutions did some kind of awareness activities which was not a direct statutory requirement, but a recommended guideline.

Conclusion

The results show that the intent to decentralise the compliance of the tobacco control law in New Delhi by the higher educational institutions has not been universally successful and requires much effort for its on-ground penetration. Such studies have a policy impact as they serve as an example for the generalisability of such statutes, which only work when there is implementation from the bottom-up and when the deterrent is also strong with incentivisation to the educational institutions to implement tobacco control with vigour.

## Introduction

India has an anti-smoking and anti-tobacco law that was passed in the year 2003. It is known as the Cigarettes and Other Tobacco Products (Prohibition of Advertisement and Regulation of Trade and Commerce, Production, Supply and Distribution) Act 2003 or the COTPA Act 2003 [1]. The intent of the law was to enact ‘a comprehensive law on tobacco in the public interest and to protect the public health’ and to ‘prohibit the consumption of cigarettes and other tobacco products which are injurious to health with the view of achieving the public health in general as enjoined by Article 47’ of the Indian Constitution [2]. This was made in response to the 43rd World Health Assembly, where member states were urged to consider tobacco control strategies, with a focus on protecting children from voluntary exposure to tobacco use and discouraging the use of tobacco [3]. It is this focus on young people that the Indian law focused on educational institutions and minors. The legal manifestation of this concern for minors and educational institution vicinity is seen in Section 6 of the COTPA Act 2003, which states that:

No person shall sell, offer for sale, or permit sale of, cigarette or any other tobacco product- (a) to any person who is under eighteen years of age, and (b) in an area within a radius of one hundred yards of any educational institution.

This clearly shows that the intent of protecting the youth from tobacco and smoking existed, even if it was in response to a national commitment in front of an international forum.

But the most important and missing link in this is the way this would be implemented. The concern was raised even in the parliament when this bill was taken in the Lok Sabha on the date of its adoption as a law passed by the legislature in India after one house of the parliament had already passed it. The concern raised was whether simple enactment of the law will actually lead to its implementation [4].

This leads us to the second decision that the government made to solve the problem of the implementation of COTPA Act for its implementation, especially with respect to banning the sale of tobacco products within 100 yards of educational institutions. The government simply outsourced and decentralised the compliance to educational institutions by enacting a rule under section 31 of the COTPA Act called the Cigarettes and Other Tobacco Products (Display of Board by Educational Institutions) Rules 2009, which states as follows [5]:

3. Display of Board by Educational Institutions.(1) The owner or manager or any person in-charge of affairs of the educational institution shall display and exhibit a board at a conspicuous place outside the premises, prominently stating that sale of cigarettes and other tobacco products in an area within a radius of one hundred yards of educational institutions is strictly prohibited and that it is an offense under Section 24 of the Act with fine which may extend to two hundred rupees.’

By this act, the government outsourced and decentralised the compliance to educational institutions and that too with the only requirement of putting up a signboard that prohibits anyone from selling cigarettes and tobacco products within 100 yards of educational institutions. There may also be scope for an assumption that putting such a board may not be deterrent enough, as even if someone has been caught, the meagre punishment of a fine of rupees 200 will not only be too little but will also be over as a summary trial. The lawmakers, while discussing it, also had similar concerns as this may not be deterrent enough, and people may pay the money as there is no severe punishment for the re-occurrence of the offence by the same person [4].

Another way by which compliance has been decentralised is by giving two more responsibilities to educational institutions. One is the installation of a board which states under the Prohibition of Smoking in Public Places Rules 2008, at multiple places, including the entrance, other conspicuous places, and one on each floor near the staircase [6]. The second is even the on-ground implementation of the prohibition of smoking in public places by making the College/School/Headmaster/Principal or even the teacher as the authorised officer to impose and collect the fine against the violation of Section 4 of the COTPA Act 2003. This means the teachers have been expected to be the law enforcers and collect fines from students who are found smoking in educational institutions. These have been reiterated in intent by the guideline of the Delhi State Tobacco Control Cell to educational institutions and are also in line with the earlier Delhi Assembly statute, which preceded the national anti-tobacco/anti-smoking Act [1,7,8].

With this, we find out that there are three responsibilities of the educational institutions by which the anti-tobacco law has to be implemented and enforced by them. These are summed up as follows, Firstly, putting a poster prohibiting the sale of cigarettes and tobacco products within 100-yard vicinity of educational institutions. Secondly, putting a board prohibiting smoking within educational institutions and thirdly, imposing and collecting the fine from anyone found smoking in educational institutions.

To check compliance for the above three points, this study has been designed at the level of the higher educational institutions in New Delhi. The aim of this study is to find the decentralised implementation of the COTPA Act 2003 and its rules by educational institutions in New Delhi. This compliance has been checked for national-level institutes by the same author [9], but the compliance on the level of Delhi, with respect to fine collection and placement of indoor posters, has not been covered fully by any other study from India or from the world for that matter. The author, in an earlier study, has looked for the checking of the signboard compliance prohibiting the sale of tobacco products within 100 yards of the vicinity but has found non-universal compliance with the signboard requirement [10]. The need for this study is justified as this may be the first study looking comprehensively at the compliance by the educational institutions in the heart of India, in its capital city of New Delhi.

## Materials and methods

This study focuses on higher education institutions. Forty-seven higher education institutions, most of them run by the Government of National Capital Territory of Delhi, or the Government of NCT of Delhi, have been included. This number is of randomly selected higher educational institutions, including four universities run by the Government of NCT of Delhi located in New Delhi and all 12 colleges of the Delhi University that are fully sponsored by the Delhi government, i.e., colleges funded 100% by the Delhi Government, but under the Delhi University [11]. Many colleges affiliated with the universities and other autonomous stand-alone colleges run directly by the Delhi Government have been included. This is a fairly representative sample, as there may be a lack of knowledge of the exact number of all the colleges run by various governments and this lack of calculation of sample size may be a limitation of the study. But covering four universities, all Delhi government-run colleges of the Delhi University provide a fairly good representation of higher educational institutions in New Delhi. The author has performed a separate study for the colleges run by the Central Government in India, which also includes the centrally run colleges in New Delhi like the Indian Institute of Technology, New Delhi [9].

This study uses a unique methodology, where the transparency law of India has been used for data collection from the various institutions that have been included in the study. The Right to Information Act of 2005, or the RTI Act 2005, usually otherwise used for holding the government to account, has also found its use in this study where it has been used for research [12,13]. This is just like the Freedom of Information Act or FOIA Act of the United States. Out of the 47, 11 institutions did not respond to the request for the information in defiance of the RTI Act 2005. This can lead to penalty and disciplinary action, and the author will be following up with the institutions as they have not responded to the request, which is not only prohibitive for research but is also illegal as per law. In this study, therefore, 36 institutions have finally been included out of the 47 that were sent applications under the RTI Act 2005.

The information asked for was in the sense of checking the decentralised application of the COTPA Act 2003 by the educational institutes. The information was asked in the form of applications filed under Section 6 of the RTI Act 2005. This mandates the public authority, which in our case is the educational institutions run by the Government of NCT of Delhi, as mentioned before, to provide the information under the seal and signature of a senior officer of the public authority, which is usually a senior faculty in the case of educational institutions. This information has to be supplied within a mandatory time period of 30 days as per Section 7(1) of the RTI Act 2005. This approach enables only authentic, duly certified and accurate information. This information, since released under the public transparency law, also becomes information in the public domain and is available for wide public usage. The limitation of using the RTI Act 2005 is twofold. Firstly, the information has not been ground-truthed by the author, and it relies on the information provided by the officer. But since the information is of legal nature, this apprehension may be diluted as the government officer will not provide false information under their sign and seal. Secondly, another limitation is that this approach is only valid for educational institutions which come under the category of public authority as per Section 2(h) of the RTI Act 2005. This automatically excluded the private educational institutions that are present in New Delhi but are not included in this study. Due to the exclusion of these under the transparency law, these private institutions become somewhat opaque and cannot be mandated by law to provide information of public interest, and researchers may need to depend on the choice and will of these institutions to provide such information, and this may lead to the provision of no information at all. This is because, due to the absence of a legal requirement, information that may put the institution in a bad light may never be available for researchers, journalists and others interested.

The educational institutions, once selected, were sent an application as mentioned before with the following points of information. Firstly, information regarding the presence of the signboard outside of the educational institution in compliance with the Cigarettes and Other Tobacco Products (Display of Boards by Educational Institutions) Rules, 2009, which instruct educational institutions to put up a board that states to prohibit the sale of cigarettes and tobacco products in an area within a radius of one hundred yards [5]. Secondly, information regarding the presence of boards and their location state that the educational institution is ‘No Smoking Area-Smoking Here is an offence’ as per Section 3(b) and Schedule II of the Prohibition of Smoking in Public Places Rules, 2008 [6]. Thirdly, information of the total instances of fines collected/offences compounded/offences recorded/warning issued in respect to Section 5 of the Prohibition of Smoking in Public Places Rules, 2008, which stated the authorised officers who are competent to act under Schedule III of the Prohibition of Smoking Rules, 2008 from the year 2012 till the year 2022. In this, the Principal/teacher/director/head of the institution is a person authorised to take action under the law. Fourthly, information on the presence and number of vendors of cigarettes and other tobacco products within a distance of 100 yards from the outer boundary of the educational institute. Lastly, information regarding the list of events/initiatives/activities/circulars/anything on record where the educational institution has taken steps to prevent the use of cigarettes and tobacco products.

An annexure with the detailed quoted laws was also annexed with the application. The institutions provided the reply through the RTI Act 2005 portal as well as written replies with signatures on the letterhead of the institution [14]. The period of collection of the information has been between April to May 2023. The information from the educational institutions was received in three ways. Some sent a detailed hard copy; others uploaded it to the RTI portal and some sent it via email. This information was collected till the end of the one-month information period and even extended by another month of grace period to cater for the postal delays or any other delays. These results were compiled, reported, and interpretation was drawn and made in line with the aims and objectives of the study. Another limitation that is reported is that the first reply by the educational institutions has been reported, and the first appeal or the second appeal has not been filed to obtain clarification on the information provided in the first reply by the educational institutions.

Non-requirement of ethics review for this study

This study uses data available in the public domain that has been supplied by a public authority under the RTI Act 2005. There is no involvement of any human participant, human subject, human or animal tissue. There is also no linked identifier to any human participant or subject. The mere fact that the data have been supplied under the open public domain through the RTI Act 2005 deems it non-personal as, according to the transparency law, the public authority cannot provide any third-party information by law.

This article was previously posted to the medRxiv preprint server on June 20, 2023.

## Results

In general, out of 36 institutions that provided the information, on the issue of the provision of the signboard prohibiting the sale of cigarettes and tobacco products within a vicinity of 100 yards, 16 (44.4%) have provided the board in the format required, which means the board stated explicitly that the ‘Sale of cigarettes and other tobacco products within 100 yards of the educational institutions is an offense …’. Seven institutions stated clearly that the board has not been provided, though, from the reply, many of the seven were in the belief that the placement of the board was not their own responsibility but was of some other agency. This will be discussed in the paper later. Seven out of the 36 stated there was a presence of the board, but it was not clear whether the format was as required, as many of these seven stated that they had placed ‘No Smoking’ boards instead of the boards which prohibit the sale within 100 yards vicinity. It is also pertinent to note that six out of the 36 that provided information, in general, did not provide the information related to the signboard prohibiting sale within 100 yards. They either stated that the information is either not on record or used some other way of denying the information.

But, with respect to the placement, the simple signboards prohibiting smoking in educational institutes as public places, the signboard compliance was more promising. Out of 36 institutions, 32 (88.9%) have the boards placed. Two institutions did not place the boards, and two did not provide the information.

On the matter of the recording instances of smoking/instances of collecting the fines, these two were together clubbed, and it was found that only one institution has a record of smoking instances/fine collection. Seventeen (47.2%) educational institutions had no instances of smoking recorded/no fines collected. For this, a staggering 18 (50%) institutions did not provide the information or did not have such information on record.

With respect to the issue of whether there was a presence of tobacco vendors within 100 yards of the educational institution vicinity, it was found that two institutions reported the presence of vendors. Seventeen on the other hand, stated that there was no presence of the vendors. Seven institutions responded that this information was either not under the purview of the educational institution or was not a responsibility of the educational institution. Some even went ahead and transferred this part of the information either to the Delhi Police or to the health department. Ten educational institutions denied the information or did not provide or stated that the information was not on record.

As education is the major role played by educational institutions in society, we checked the role played by educational institutions in educating about tobacco cessation. We found that out of 36 educational institutions, 27 stated that they conducted activities related to educating students on tobacco cessation. These activities may range from counselling to plays, dramas, talks, and other seminars. One institution reported no activities on tobacco cessation, and eight did not provide any information and stated that no such information was on record. The results stated as above have been summarised in Table 1.

**Table 1:** A summarised version of the results showing the compliance by 36 educational institutions on the various tobacco control requirements. N.A.: Not Applicable

S. No.	Nature of compliance for tobacco control	No. of institutions that complied	No. of Institutions where the compliance was not complete or there was lack of clarity	No. of institutions that did not comply	No. of institutions that did not provide information about this compliance	Remarks
1	Installation of board prohibiting sale of cigarettes and other tobacco products within 100 yards of the educational institution in the proper format.	16	7; The board was not explicitly stated to be in the format required, with some providing No Smoking boards instead.	7	6	Many of the seven that did not provide the board were in the misbelief that the placement of the board was not their own responsibility but was of some other agency like the police or the health department.
2	Signboards prohibiting smoking in educational institutions as a public place.	32	0	2	2	
3	Recording of instances of smoking/instances of collecting the fines	1; Record of smoking/fine collection. 17; No instances of smoking recorded/No instances of fines collection.	N.A.	None stated explicitly.	18	A staggering 18 institutions did not provide the information or did not have such information on record. This directly translates to non-compliance of the law.
4	Presence of tobacco vendors within 100 yards of the educational institution.	2; reported presence of such vendors. 17; Reported non presence of vendors.	7; Stated that the information was neither under the purview of the educational institution nor was a responsibility of the educational institution	N.A.	10	Some educational institutions transferred this particular point to be provided information by the Delhi Police, as their responsibility.
5	Conducting activities for education on Tobacco Control and Cessation	27	0	1	8	This is not particularly a statutory compliance.

## Discussion

This study is not the first one to check the compliance of the COTPA Act 2003 in India, yet it is unique and brings forth new knowledge in new ways. The basis of the study has been the decentralisation of the compliance of the COTPA Act 2003 in educational institutions of New Delhi, India, which forms the heart of the second most populated country in the world. The first contribution of this study is that it is the first one which checks the decentralised compliance of educational institutions in Delhi with respect to the detail of the smoking instances checked or fines collected. This is something that is part of the law, where the decentralised duty is given to the principal, headmaster, and faculty of the educational institution, but this check is not part of the standard check that forms part of the ‘Self-Evaluation Scorecard for Tobacco Free Educational Institution’ released by the Ministry of Health and Family Welfare, Government of India [15]. There are also other studies that are related to compliance with the COTPA Act 2003 in India. The first study is which only simply measured advertisements at vendor’s points and only selected 30 schools randomly [16]. This study did not include signboard compliance or fines compliance. Another group performed two studies performed cross-sectional and proximity analysis [17,18]. The group earlier did a multi-stage cluster sampling survey for checking the advertisement by tobacco vendors. In these, the signboard component has been missed. Another study used questionnaires for compliance. But even this study was restricted to the ‘No Smoking boards’ compliance and did not include the signboard prohibiting sale in 100 yards vicinity [19]. Another study performed concentrated on tobacco vendors on various parameters, including advertisement and selling the products to minors [20]. This current study is useful as it deals with three important points related to two formats of signboard compliance and the third being the compliance of smoking instance/fine collection recording. The author has himself performed a similar study on national-level educational institutions and also performed ground-truthing of the signboard compliance (the board banning sales within 100 yards) and the actual presence of the vendors within 100 yards of the educational institutions [10]. Another unique component of this study is the sourcing of the data right from the source itself. Using the transparency law of India, i.e., the RTI Act 2005, ensures that the information provided is most authentic as it has been provided under the sign and seal of the educational institution under a legal requirement, with penal punishments available for the provision of wrong information.

There is also the important issue of the seeming confusion regarding the format of the boards. There were seven institutions that did not provide the appropriate reply to the issue of the board prohibiting the sale of cigarettes and tobacco products within 100 yards of the educational institutions. Some institutions seemed to be using the two boards interchangeably, where the ‘No smoking’ boards were only represented as the board which prohibited the sale of cigarettes within 100-yard proximity. This has also been confirmed in another context by another ground-truthing study by the author in Delhi [10].

Another issue of important concern is the belief that some educational institutions had regarding the two issues, where the educational institutions assumed that the duty did not lie with the educational institution but with some external agency like the police or the health department of the government. The first is the placement of the board prohibiting the sale of tobacco within 100 yards of the educational institutions, where the educational institutions in question, stated that this was the responsibility of the police or the government agencies to place the boards. This belief is not only false but is also illegal and in direct contravention of the COTPA Act 2003 and its related rules. Another important factor that the educational institutions believed are not ‘under the purview’ of the educational institution is the presence of cigarettes and tobacco products vendors within 100 yards of the educational institution's vicinity. This may appear true in the first instance, but from the point of view of decentralisation, it may then be an educational institute that may either be the direct fine imposing authority or maybe the first responder by informing the law enforcement agencies. But, from the point of view of the guidelines issued by the Ministry, as stated before, one of the important criteria for self-evaluation for check compliance of the COTPA Act 2003 is the check for the presence of tobacco vendors within proximity of 100 yards. By the presence of this factor in a ministry-issued guideline, educational institutions cannot absolve themselves of the responsibility of checking for the vendors selling cigarettes and tobacco products and reporting the same to law enforcement agencies [15]. This has not been followed by the educational institutions that have in the information provided seemingly outsourced this responsibility to either the police or the health department.

As another peripheral issue, the awareness activities organised by the institutions are also an important step by the educational institutions with 75% of the institutions undertaking such activities. This is not a direct statutory requirement but is part of the guidelines issued for educational institutions and is an important duty of educational institutions for tobacco control and cessation [7,15].

Another important issue mentioned earlier, deals with the collection of fines. The empowerment of the headmaster, principal/head of the institute, or even the faculty member to impose and collect the fines is a decentralised approach to the compliance of the COTPA Act 2003 by the government, made in response to the legislature’s wish of implementing the act. At first glance, it may appear as a very positive step as the power has been transferred to the actual person on the ground till the last mile, but at the same time, it appears that there may also be concerns regarding the outsourcing of responsibility to the educational institutions.

The study presents a picture of the situation of compliance with the tobacco control law in Delhi by educational institutions. But there are a few limitations that have been mentioned before but must be summarised. The first is the lack of calculation of a sample size for this study which includes only 36 institutions that provided the information. The second limitation is that the ground-truthing of the information provided by the educational institution has not been done and it relies on the information provided under seal by the educational institution (though as a legal obligation, where false information may not be provided). The third limitation is that the methodology of collection of information is valid only for government institutions as the RTI Act 2005 application can only be filed before a public authority, which means a government-run or government-sponsored institution. This excludes private institutions from the study. The fourth limitation of the study has been that the first reply of the educational institution has been studied and used in the study. The legal option of a first appeal or second appeal has not been used to either seek clarification on the information provided or to demand information from the institutions that did not provide a reply. Though, the first reply was sufficient for this study. The last limitation in this study is the expandable scope not covered in the study like advertisements of tobacco products.

From the generalisability point of view, it is important to understand that making a statute and effecting its implementation are important points to consider. There may be other such statutes that prohibit speed limits, fast food sales, or any other prohibition which may be top-down in nature. For the statute to actually be implemented, capacity buildings, training, and regular outreach must be part of the process in order to make the implementation more bottoms up in nature.

Future scope

There is also scope for further study in multiple areas, including the implementation check in all geographies. There is also further scope of another issue of research as to whether 100 yards limit is effective and whether this needs to be increased, and whether the simple position of a signboard by an educational institution is deterrent enough for the vendors to not sell cigarettes and tobacco within the vicinity of educational institutions. Also, to check whether the process of imposing fines is an effective measure as a person affording a cigarette may pay the fine and carry on with smoking due to lack of stricter punishments or on recurrence of the offence. There is further scope to check the quantum of the fines collected, and a pilot study for implementation. Studies looking into the linking of compliance to counselling and the promotion of steps like an anti-tobacco helpline should be performed. Studies related to the advertisement of tobacco products can also be performed.

## Conclusions

The aim of this study is to find the decentralised implementation of the COTPA Act 2003 and its rules by educational institutions in New Delhi. As far as the installation of boards prohibiting the sale of cigarettes and other tobacco products is concerned, the implementation is not universal, with only 44.4% of the institutions having a board. In the case of the no-smoking board, compliance was better, with 88.9% of the institutions having such boards. On instances of smoking and fine collection, only one institution reported an instance and collected a fine, while 47.2% of institutions did not have any instance of smoking/collected fine. It is negative that about 50% institutions did not even have a record of this collection of fines and recording of the smoking instances, which can safely be assumed as defiance of the law. In New Delhi, with the sample size studied, it can be stated that compliance with the COTPA Act 2003 is not universal in the complete sense, and there are many areas that need attention. The COTPA Act 2003 must be made stricter with more severe and deterring punishments for the offenders. The police should coordinate with educational institutions by taking the lead and initiative so that the vendors selling cigarettes and tobacco products within 100 yards can be punished severely.

The intent of decentralising the role to the educational institutions has not been universally implemented, with some educational institutions not even owning up to the fact that the compliance of installing a signboard prohibiting the sale of cigarettes in the vicinity of 100 yards falls in their responsibility. A push needs to be made to incentivise educational institutions in the form of academic accreditations and enhanced academic rankings if they are able to fully implement the COTPA Act 2003 in and around their premises.
